# Was it a vision or a waking dream?

**DOI:** 10.3389/fpsyg.2014.00255

**Published:** 2014-04-04

**Authors:** Robin Carhart-Harris, David Nutt

**Affiliations:** Division of Brain Sciences, Department of Medicine, Centre for Neuropsychopharmacology, Imperial College LondonLondon, UK

**Keywords:** consciousness, REM sleep, dreaming, hallucinogens, posterior cingulate cortex, default mode network

*Was it a vision, or a waking dream?* (John Keats, 1795–1821. Ode to a Nightingale).

Reminiscent of Wilder Penfield's famous experiments, Neurologists in France have reported a remarkable case in which intraoperative electrical stimulations of the posterior cingulate cortex (PCC) in a conscious patient induced transient dreamlike states with vivid visual imagery (Herbet et al., 2014). The implicated circuitry and nature of the experiences evoked comparisons with findings from our own neuroimaging research with the hallucinogen and putative “oneirogen” (dream-inducer) psilocybin, strengthening what can be inferred about the importance of the PCC in mediating the quality of consciousness.

We were fascinated to read the case-report of a dreamlike experience evoked by direct electrical stimulation of the posterior cingulate cortex (PCC) in an epilepsy patient by Herbet et al. (2014). The PCC has attracted a lot of interest in recent years due to recognition of its high metabolic and vascular demand (Raichle et al., 2001) and importance as a cortical connector hub (Hagmann et al., 2008) and integration center (Leech et al., 2012). Perhaps due to its buffered location and rich vascular innervation, there is an absence of cases of focal PCC lesions (Leech and Sharp, 2014) and to our knowledge there are no reports on the effects of PCC stimulation in humans. There are a few case-reports of impaired spatial navigation and related symptoms of Balint's syndrome in patients with damage to the retrosplenial cortex (Leech and Sharp, 2014) but the stimulation site here was dorsal to the retrosplenial cortex, in white matter of the cingulum bundle, a major tract connecting the PCC with the medial prefrontal cortex (mPFC). This circuit constitutes the spine of the default-mode network (DMN), a system that has been associated with spontaneous cognition that is suspended or interrupted during periods of externally-directed attention (Raichle et al., 2001).

Upon reading Herbet et al.'s report, we were struck by similarities between the subjective reports given post-PCC stimulation and those we observed after controlled administration of the classic hallucinogen, psilocybin (Carhart-Harris et al., 2012; Muthukumaraswamy et al., 2013). As Herbet et al. discuss, stimulation of the PCC/cingulum bundle likely inhibited activity in this region and interrupted communication between the mPFC and PCC. Importantly, altered PCC activity (i.e., decreased blood flow and oscillatory power and mPFC-PCC functional connectivity) was the most conspicuous and reliable finding of our psilocybin imaging studies and volunteers reported experiencing a dreamlike state and vivid visual imagery (Carhart-Harris et al., 2012, 2014; Muthukumaraswamy et al., 2013). Moreover, sustained improvements in well-being (Griffiths et al., 2006) and lasting decreases in depressive symptoms have been reported post-psilocybin (Grob et al., 2011) and it was remarkable to read Herbet et al.'s patient describe an absence of rumination and “absolute happiness” for a sustained period after resection of the PCC. Is it possible that psilocybin produces a sustained alteration in PCC and/or DMN activity that could account for its putative therapeutic potential (Carhart-Harris et al., 2014)? This is something we intend to test in a forthcoming trial of psilocybin as a treatment for major depression (Roiser and Rees, 2012).

Finally, the theoretical implications of Herbet et al.'s report are profound. The authors note that PCC cerebral blood flow is decreased in rapid eye movement (REM) sleep relative to waking (Braun et al., 1997) and non-REM sleep (Maquet et al., 1996). It has long been a matter of intrigue to us that LSD given just before sleep onset (Muzio et al., 1966) or intravenously during sleep (Torda, 1968) markedly promotes REM sleep. The classic serotonergic hallucinogens LSD, psilocybin and dimethyltryptamine are known to produce vivid and complex imagery, especially with eyes-closed, that are often described as dreamlike (Grinspoon and Bakalar, 1979). Another common feature of the REM-sleep and hallucinogenic drug states is alterations in medial temporal lobe (MTLs) activity. For example, the MTLs are hyperactive in REM-sleep (Maquet et al., 1996; Braun et al., 1997; Miyauchi et al., 2009) and show an increased amplitude in their signal fluctuations post-psilocybin (Carhart-Harris et al., 2014) which correlates with reports of dreamlike phenomena (Figure 1). The MTLs are another area where electrical stimulation can produce vivid dreamlike visions of the sort reported by Herbet et al.'s patient (Vignal et al., 2007). MTL stimulations producing dreamlike states have been found to induce a spreading activation from the stimulation site to the temporal and visual cortex (Barbeau et al., 2005; Bartolomei et al., 2012). However, the stimulations in the present case were in white matter and thus likely had an inhibitory rather than excitatory effect (Holtzheimer et al., 2012).

**Figure 1:**
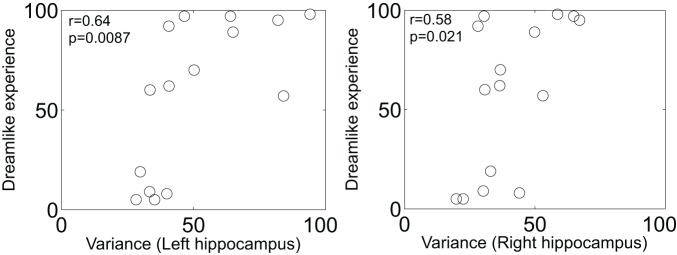
**Scatter plot of the increase in left and right hippocampal blood oxygen-level dependent signal variance/amplitude (after psilocybin infusion) vs. subjective ratings of the item: “the experience had a dreamlike quality.”** Both measures are expressed as a percentage increase from baseline. There were no changes in BOLD signal variance after placebo infusion (*n* = 15) (Carhart-Harris et al., 2014).

So, how might disrupting DMN activity be the cause of dreamlike visions? One way to address this question is to look for clues from studies on REM-sleep and other dreamlike states. Firstly, it is worth noting that the MTLs are major subcortical nodes of the DMN (Supekar et al., 2010) but under psilocybin, MTL-DMN coupling is decreased (Carhart-Harris et al., 2014). Similarly, PCC activity is decreased in REM-sleep (Maquet et al., 1996) and under psilocybin (Carhart-Harris et al., 2014) but MTL activity is increased (Maquet et al., 1996; Carhart-Harris et al., 2014). Since MTL activity is coupled to phasic events in REM-sleep, including REMs (Bodizs et al., 2001; Karashima et al., 2007), the link between MTL activity and dreaming appears to be particularly intimate. Thus, disrupting DMN activity may have had a disinhibiting effect on MTL activity (Carhart-Harris et al., 2014) and this may have been the cause of the ensuing dreamlike visions (Carhart-Harris, 2007; Carhart-Harris and Friston, 2010; Carhart-Harris et al., 2014). Future neuroimaging work on dreaming and dreamlike states will help to inform these speculations.

In summary, we were struck by similarities between Herbet et al.'s findings with direct electrical stimulation of the PCC and those of our own with psilocybin and neuroimaging. Their case provides some causative support for the notion that the PCC is centrally involved in mediating the *quality* of consciousness (Carhart-Harris et al., 2014), and more specifically, that inhibiting the PCC/disrupting DMN activity can induce dreamlike states. Moreover, we are intrigued by the possibility that drug and stimulation-induced dreamlike states are indeed truly dreamlike, i.e., in the neurophysiological sense as well as the phenomenological.

## Conflict of interest statement

The authors declare that the research was conducted in the absence of any commercial or financial relationships that could be construed as a potential conflict of interest.
